# Enhanced GABAergic actions resulting from the coapplication of the steroid 3α-hydroxy-5α-pregnane-11,20-dione (alfaxalone) with propofol or diazepam

**DOI:** 10.1038/s41598-018-28754-7

**Published:** 2018-07-09

**Authors:** Lily Q. Cao, Michael C. Montana, Allison L. Germann, Daniel J. Shin, Sampurna Chakrabarti, Steven Mennerick, Carla M. Yuede, David F. Wozniak, Alex S. Evers, Gustav Akk

**Affiliations:** 1 Departments of Anesthesiology, Washington University School of Medicine, St. Louis, MO 63110 USA; 2 Psychiatry, Washington University School of Medicine, St. Louis, MO 63110 USA; 3 Neurology, Washington University School of Medicine, St. Louis, MO 63110 USA; 4 The Taylor Family Institute for Innovative Psychiatric Research, Washington University School of Medicine, St. Louis, MO 63110 USA

## Abstract

Many GABAergic drugs are in clinical use as anesthetics, sedatives, or anxiolytics. We have investigated the actions of the combinations of the neuroactive steroid 3α-hydroxy-5α-pregnane-11,20-dione (alfaxalone) with the intravenous anesthetic propofol or the benzodiazepine diazepam. The goal of the study was to determine whether coapplication of alfaxalone reduces the effective doses and concentrations of propofol and diazepam. Behavioral effects of alfaxalone, propofol, diazepam, and the combinations of the drugs were evaluated during a 30-min activity test in mice. Functional effects of the individual drugs and drug combinations were tested by measuring the decay times of spontaneous inhibitory postsynaptic currents in rat hippocampal neurons, and peak current responses from heterologously expressed concatemeric α1β2γ2L GABA_A_ receptors. Co-administration of alfaxalone increased the sedative actions of propofol and diazepam in mice. The combination of alfaxalone with propofol or diazepam increased the decay times of sIPSCs and shifted the concentration-response relationships for GABA-activated receptors to lower transmitter concentrations. We infer that alfaxalone acts as a co-agonist to enhance the GABAergic effects of propofol and diazepam. We propose that co-administration of alfaxalone, and possibly other neuroactive steroids, can be employed to reduce dosage requirements for propofol and diazepam.

## Introduction

The γ-aminobutyric acid type A (GABA_A_) receptor is the principal inhibitory ionotropic transmitter-gated ion channel in the brain^1^. A number of GABAergic compounds are in clinical use as anesthetics, sedatives, anxiolytics, and/or anticonvulsants^2,3^. These include intravenous anesthetics, such as propofol, and benzodiazepines, such as diazepam. Propofol is used for induction and maintenance of general anesthesia of surgical patients as well as sedation of critically ill patients. Diazepam is first-line treatment for seizures, and is also used to treat anxiety and panic attacks. Both drugs are widely-used clinically but can have significant shortcomings due to undesired side effects. Propofol can cause hypotension and respiratory depression at plasma concentrations reached during anesthesia^4,5^. Diazepam, particularly at higher doses, results in anterograde amnesia and sedation^6,7^. Thus it could be advantageous to develop approaches to administer lower doses of the drugs while maintaining overall clinical efficacy.

One conceivable approach to reduce drug dosage without compromising the desired clinical effects is to combine the clinical agent with another GABAergic compound, such as a potentiating neuroactive steroid, that acts as a background agonist or a co-agonist in activating the GABA_A_ receptor. A recent study showed that the neurosteroid 3α-hydroxy-5α-pregnan-20-one (allopregnanolone) augments the ability of propofol to reduce action potential firing and prolong inhibitory postsynaptic currents in neocortical neurons^8^. We have previously shown that etomidate-mediated potentiation of the recombinant α1β2γ2L GABA_A_ receptor is amplified in the presence of the neurosteroid 3α-hydroxy-5β-pregnan-20-one (pregnanolone). The potential clinical relevance of the effect was demonstrated in behavioral studies, which revealed left-shifted dose-response relationships for loss-of-righting by etomidate in mice and *Xenopus* tadpoles, in the presence of the neurosteroid^9^.

To further explore this phenomenon, and to determine how it manifests in various *in vivo* and *in vitro* experimental assays, we investigated the behavioral and functional effects of co-administration of the synthetic neuroactive steroid 3α-hydroxy-5α-pregnane-11,20-dione (alfaxalone) with propofol or diazepam. We employed alfaxalone because of its low logP value and relatively fast washout^10^. We show that the combination of alfaxalone with propofol or diazepam enhances sedation in mice as assessed by quantifying lack of movement during a 30-min activity test conducted immediately following drug administration. Animals injected with the combination of propofol and alfaxalone also demonstrated loss-of-righting in the majority of animals whereas administration of either drug alone was ineffective. Coapplication of alfaxalone with propofol or diazepam increased the decay times of spontaneous inhibitory postsynaptic currents (sIPSCs) in rat hippocampal neurons. In two-electrode voltage clamp recordings from heterologously expressed concatemeric α1β2γ2L GABA_A_ receptors, coapplication of alfaxalone with propofol or diazepam shifted the GABA concentration-response curves to lower transmitter concentrations in a manner consistent with energetic additivity. Overall, the data indicate that the GABAergic effects of propofol and diazepam can be amplified by the steroid alfaxalone.

## Results

### The effects of the steroid alfaxalone on propofol- or diazepam-induced sedation in mice

**Figure 1:**
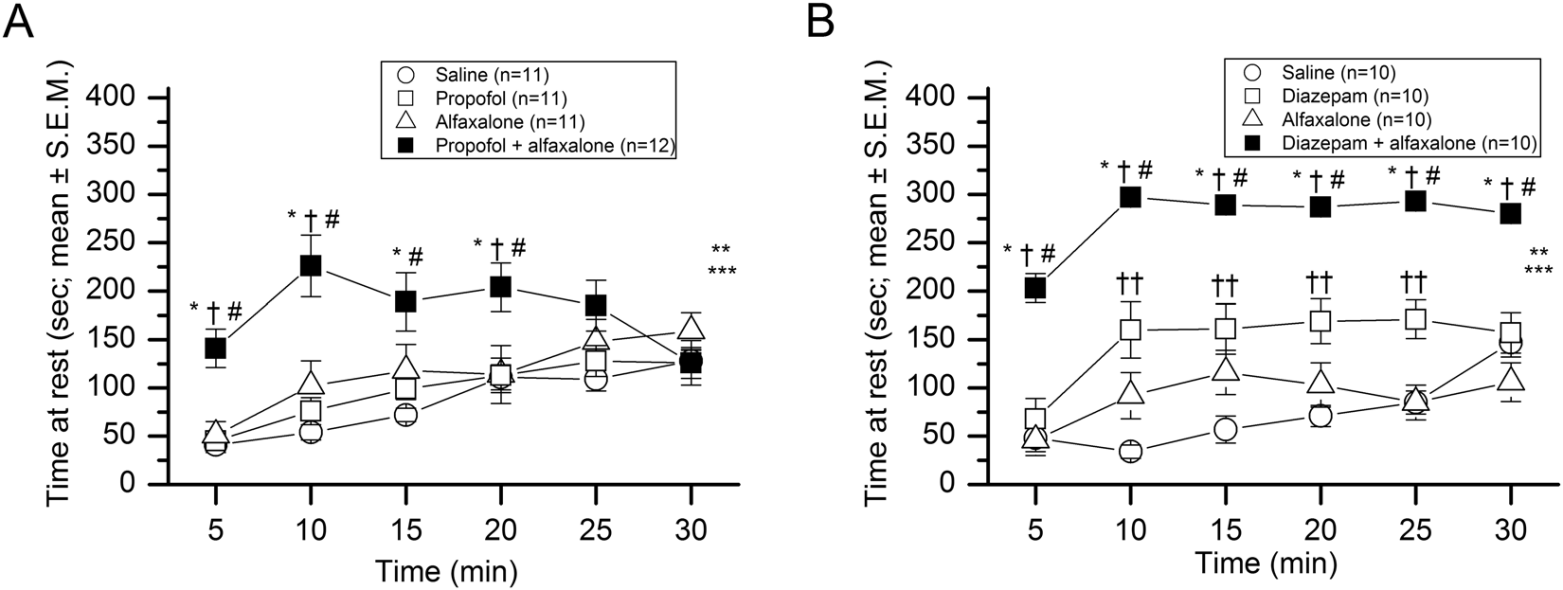
Effects of combining alfaxalone with propofol, or with diazepam on mouse behavior. (**A**) A 30-min activity test was conducted immediately following i.p. injections of normal saline (20 Units), propofol (50 mg/kg), alfaxalone (30 mg/kg), or the combination of propofol (50 mg/kg) + alfaxalone (30 mg/kg), to determine drug effects on time at rest (absence of movement) as a function of post-injection time (5-min blocks) across the test session (means ± S.E.M.; n = 11–12 mice per treatment group). A repeated measures (rm) ANOVA revealed a significant drug effect (**p = 0.0005) and drug × time interaction (***p < 0.00005), with the propofol + alfaxalone mice exhibiting significantly increased time at rest compared to the single drug/saline control groups on average across the test session. Significant between-groups comparisons are shown for these contrasts: saline vs. propofol + alfaxalone at 5, 10, 15 (*p < 0.003), and 20 (*p = 0.031) min post-injection; alfaxalone vs. propofol + alfaxalone at 5, 10 (^†^*p* < 0.002), and 20 (^†^*p* = 0.038) min; and propofol vs. propofol + alfaxalone at 5, 10 (^#^*p* < 0.0002), and 15 and 20 (^#^*p* < 0.040) min. No significant differences were observed for the contrasts involving the saline vs. propofol or saline vs. alfaxalone groups. (**B**) A separate study was conducted on an independent cohort of naïve mice to examine the potential sedating drug effects following i.p. injections of normal saline (20 Units), diazepam (4 mg/kg), alfaxalone (30 mg/kg), or the combination of diazepam (4 mg/kg) + alfaxalone (30 mg/kg) on time at rest (n = 10 for each group). An rmANOVA yielded a significant drug effect (**p < 0.00005) and drug × time interaction (***p = 0.0004), with the diazepam + alfaxalone treated mice having significantly greater times at rest compared to the saline, alfaxalone, and diazepam groups on average across the session. Between-groups comparisons conducted within these contrasts showed robust differences between each of the single drug/saline control groups relative to the diazepam + alfaxalone treated mice for every post-injection time (5–30 min) interval (*p* < 0.0002; for the comparisons involving saline (*), alfaxalone (†), or diazepam (#), respectively). The diazepam group also had significantly increased rest times relative to the saline control mice, with pair-wise comparisons revealing significant differences at 10, 15, 20, and 25-min post-injection (^††^*p* < 0.003).

Time at rest (absence of movement) was recorded during a 30-min locomotor activity test to evaluate potential sedative effects following intraperitoneal injections of alfaxalone (30 mg/kg), propofol (50 mg/kg), the combined drug treatment (propofol + alfaxalone) or normal saline. A repeated measures (rm) ANOVA on the time at rest data (Fig. 1A) revealed a significant drug effect, [F(3,41) = 7.27, *p* = 0.005) and drug × time interaction [F(15,205) = 4.16, *p* < 0.00005], indicating significant differences among some of the groups, although these differences varied across the test session. Subsequent simple main effects tests showed that the time at rest for the mice treated with propofol + alfaxalone was significantly greater (Bonferroni corrected for 5 comparisons) on average across post-injection time during the test session than those of the saline control [F(1,41) = 18.09, *p* = 0.0005], alfaxalone alone [F(1,41) = 8.39, *p* = 0.030], or propofol alone [F(1,41) = 13.64, *p* = 0.003], groups. Analyzing differences between groups as a function of post-injection time showed that the effect of the propofol + alfaxalone treatment on time at rest occurred fairly rapidly but was not particularly long-lasting. Specifically, significantly increased rest times were observed in the propofol + alfaxalone treated mice by 5-min post-injection compared to levels displayed by each single drug treatment and saline control groups, and these differences remained significantly different up until and including the 20-min time block (see Fig. 1A for “*p*” values associated with the pair-wise comparisons for specific 5-min time blocks within each of these three contrasts). Lastly, our results also showed that each of the single drug-treated groups did not differ from those of the saline control mice across the post-injection time.

A similar analysis was conducted on mice injected (ip) with diazepam alone (4 mg/kg), alfaxalone alone (30 mg/kg), the combination of diazepam + alfaxalone, or normal saline (Fig. 1B). An rmANOVA performed on the time at rest data revealed a significant drug effect, [F(3,36) = 55.68, *p* < 0.00005] and drug × time interaction [F(15,180) = 3.24, *p* = 0.0004]. Simple main effects tests showed that the diazepam + alfaxalone group displayed significantly increased rest times compared to saline control [F(1,36) = 136.12, *p* < 0.00005], alfaxalone alone [F(1,36) = 113.36, *p* < 0.00005], or diazepam alone [F(1,36) = 54.33, *p* < 0.00005] treated mice on average across the time blocks. Our analyses on group performance as a function of post-injection time indicated that the effect of the diazepam + alfaxalone treatment on time at rest occurred fairly rapidly and was long-lasting. This was documented by increased rest times in the diazepam + alfaxalone treated mice by 5-min post-injection compared to levels displayed by each single drug treatment and saline control groups. These differences remained significantly greater throughout the entire 30-min test session (see Fig. 1B for specific “*p*” values associated with the pair-wise comparisons for the 5-min time blocks). In addition, the diazepam alone group had significantly increased rest times relative to the saline control mice [F(1,36) = 18.46, *p* = 0.0006] (Fig. 1B), with significant pair-wise comparisons being observed for the 5–25 post-injection time blocks (see Fig. 1B for the “*p*” values associated with these comparisons).

**Figure 2:**
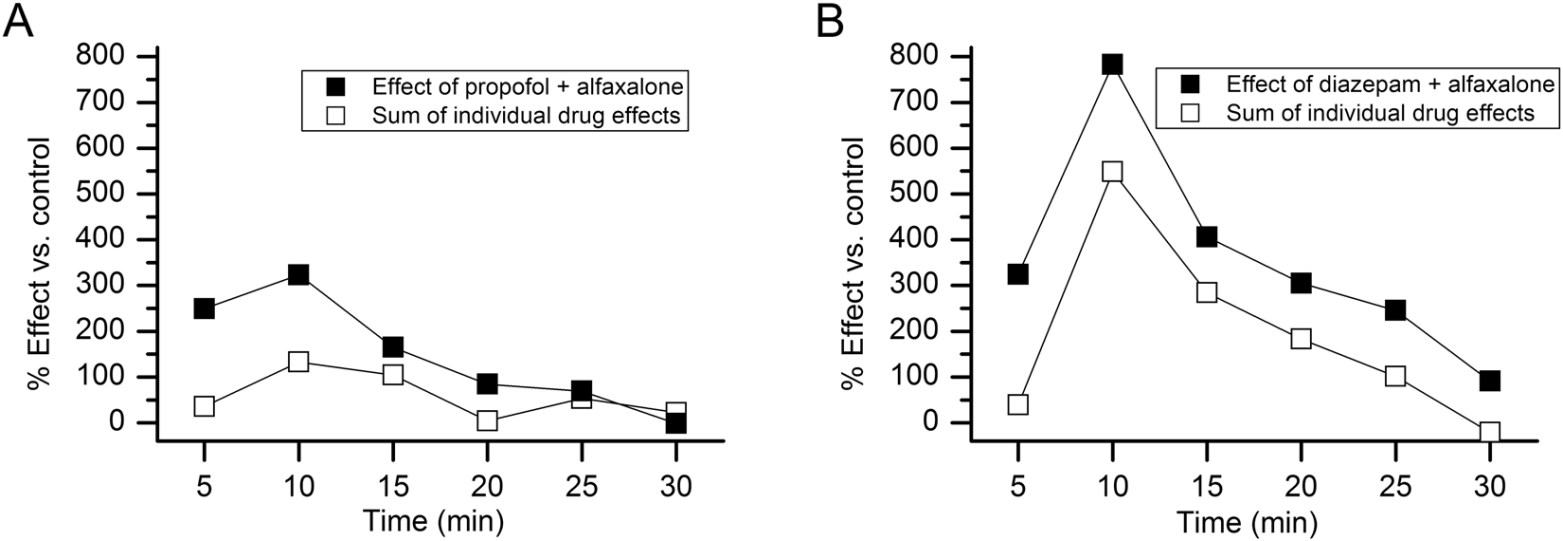
Comparison of the behavioral effects of from the co-administration of drugs with the sum of effects of individual drugs. (**A**) The graph compares the performance levels induced by the co-administration of propofol + alfaxalone to that resulting from the mathematical sums of the single drug applications. The magnitude of change in the mean percent times at rest above baseline for the co-administration of the drugs was greater early on in the test session, being approximately 7 times higher during the first time interval compared to that derived from adding together the percentages of the single applications of propofol and alfaxalone. These differences declined over the session such that the percentages from the two conditions were roughly equivalent by the end of the test session. (**B**) The graph compares the performance levels induced by the co-administration of diazepam + alfaxalone to that resulting from the mathematical sums of the single drug applications. The percent time at rest above baseline was consistently higher throughout the test session in the co-administered group compared to the percent change observed in the summed, single drug applications. The degree of change in the percent times at rest above baseline ranged from being 8.3 to 1.4 times higher in the co-administered drug group.

To provide additional perspective concerning the magnitudes of the behavioral effects resulting from the administration of propofol + alfaxalone or diazepam + alfaxalone, we compared the performance levels induced by these treatments with those resulting from the mathematical sums of the respective single drug applications. For this purpose, we computed the percent change in the mean times at rest for each of the individual and combined drug treatments by normalizing them to the mean percentages observed in the saline-injected controls from each of the two studies. We then summed the percentages from each of the single drug treatments and plotted them, along with percentages derived for the co-administered groups, as a function of time across the test session. The data from the study involving the co-administration of propofol + alfaxalone (Fig. 2A) showed that, at the beginning of the test session, the percent time at rest above baseline was approximately 7 times higher for the co-administered group compared to that derived from adding together the percentages of the single applications of propofol and alfaxalone. These differences declined over the session such that the percent time at rest above baseline was slightly greater in the combined individual drug application group compared to those of the co-administered group by the last time interval. In contrast, the data from the co-administration of diazepam + alfaxalone study (Fig. 2B) revealed that the percent time at rest above baseline was higher in the co-administered group compared to those observed in the summed, single drug applications throughout the test session. The co-administered group exhibited increased percent times at rest that ranged from 8.3 times higher during the first 5-min interval to a low of a 1.4-fold difference at 15 min. Taken together, the percent change in the time spent at rest above baseline for the co-administered drug groups supports the general idea that the sedating effects of the drug combinations exceeded the additive effects of the sum of the individual drug applications at least for some of the earlier post-injection time intervals.

The mice were also scored for loss-of-righting. A loss of the righting reflex was observed in 10/12 mice in the propofol + alfaxalone group, while it was not observed in any mice (0/11) from the other three groups treated with saline, propofol or alfaxalone. In the diazepam + alfaxalone group, loss-of-righting was observed in 2/10 mice. In saline- or alfaxalone-treated groups no mice (0/10) exhibited loss of righting whereas 1/10 mice treated with diazepam alone demonstrated loss of the righting reflex. We note that there is some apparent discordance between the time at rest and the loss-of-righting data across the two drug studies. In the propofol + alfaxalone study, high levels of time at rest and loss-of-righting were both observed in the co-administered drug group. In contrast, in the diazepam + alfaxalone study, loss-of-righting was observed in only 20% of the mice in the co-administered drug group, although the mice in this group typically displayed high levels of time at rest throughout most of the test session. The latter finding was due to most mice in the co-administered group exhibiting a post-injection response of not moving for extended periods of time, although many of them did not roll over on their sides or backs and therefore were not scored as showing a loss-of-righting.

### The effects of propofol, diazepam, and alfaxalone on the decay time courses of sIPSCs

**Figure 3:**
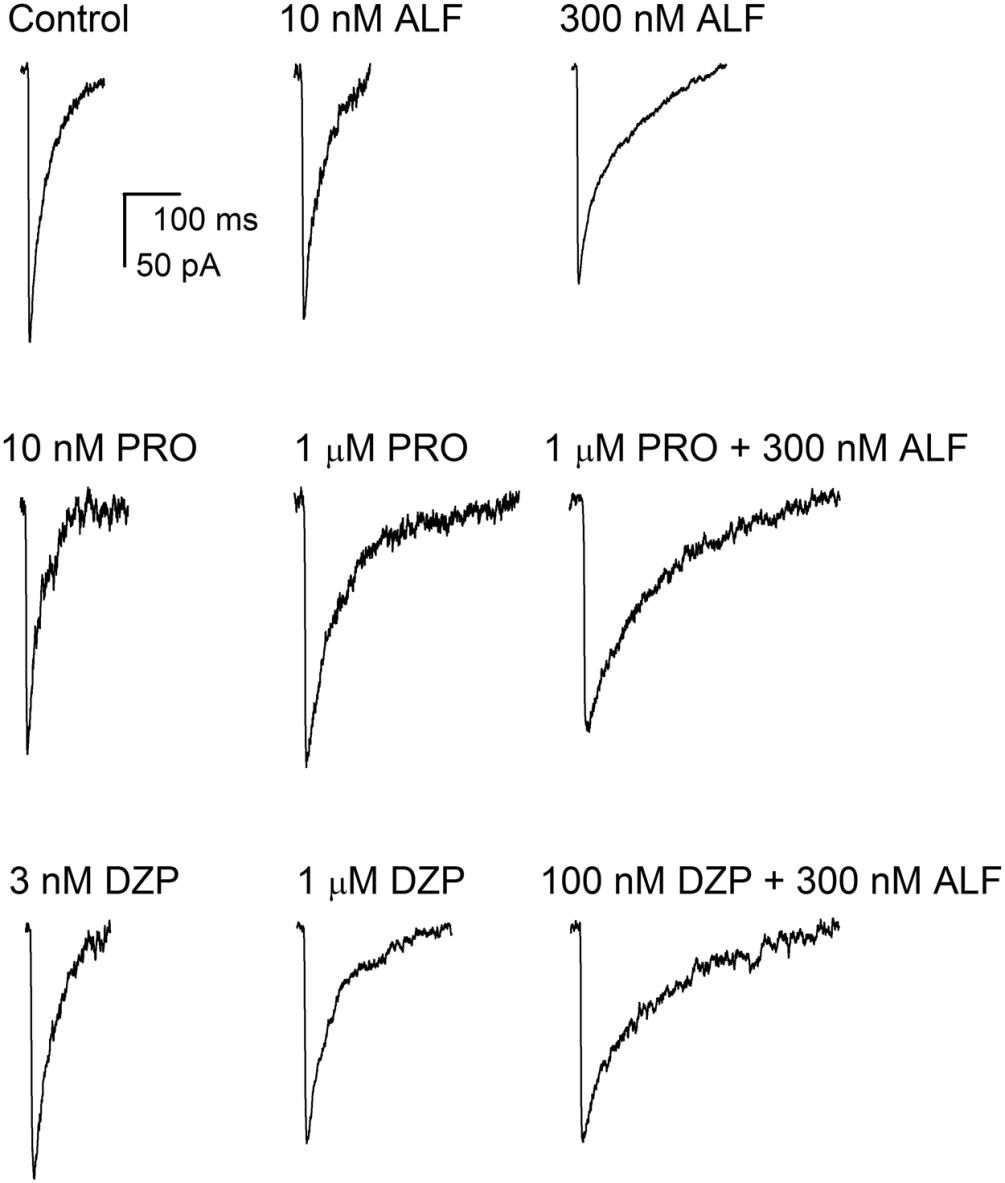
Effects of alfaxalone, propofol and diazepam on synaptic currents. Sample averaged sIPSCs recorded under control conditions, or in the presence of 10 nM alfaxalone (ALF), 300 nM alfaxalone, 10 nM propofol (PRO), 1 μM propofol, 3 nM diazepam (DZP), 1 μM diazepam, or the combinations of propofol + alfaxalone or diazepam + alfaxalone. The drugs were added to the bath at least 10 min before recordings. All traces are from separate cells.

We tested the effect of coapplication of alfaxalone with propofol or diazepam on the decay time course of sIPSCs. Under control conditions, i.e., in the absence of GABAergic modulators, the mean 100-to-25% decay time (τ_D_) of sIPSCs from cultured rat hippocampal neurons was 41 ± 9 ms (mean ± S.D.; n = 17 cells). A sample averaged sIPSC is shown in Fig. 3.

**Figure 4:**
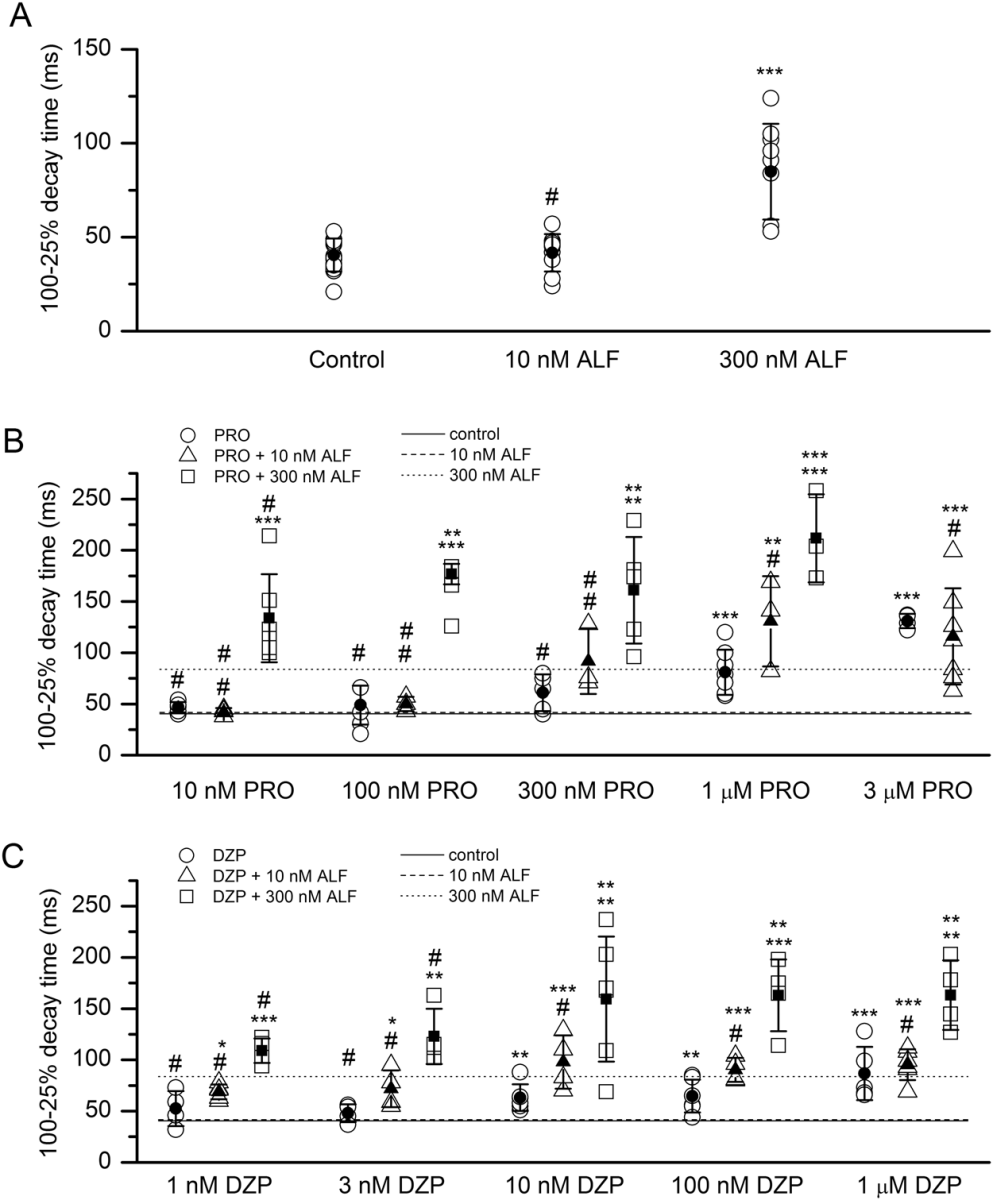
Summary of the effects of alfaxalone, propofol and diazepam on synaptic currents. (**A**) Summary of the decay time courses of sIPSCs recorded under control conditions or in the presence of 10 nM or 300 nM alfaxalone. The graph shows data from each cell tested (open circles) and mean ± S.D. for the experimental condition (filled circles and error bars). (**B**) Summary of the decay time courses of sIPSCs recorded in the presence of propofol with or without alfaxalone. The open symbols show data from each cell tested, and the filled symbols and error bars show mean ± S.D. Due to saturation at lower propofol concentrations, 3 μM propofol was not tested in the presence of 300 nM alfaxalone. Statistical analysis was done by comparing the decay times for 10 nM-3 μM propofol to control, using ANOVA with Dunnett’s correction. For combinations of propofol and alfaxalone, the top symbol applies to comparison of propofol + alfaxalone with alfaxalone alone. The bottom symbol applies to comparison of propofol + alfaxalone with propofol alone. (**C**) Summary of the decay time courses of sIPSCs recorded in the presence of diazepam with or without alfaxalone. The open symbols show data from each cell tested, and the filled symbols and error bars show mean ± S.D. Statistical analysis was done by comparing the decay times for 1 nM-1 μM diazepam to control. For combinations of diazepam and alfaxalone and, the top symbol applies to comparison of diazepam + alfaxalone with alfaxalone alone. The bottom symbol applies to comparison of diazepam + alfaxalone with diazepam alone. In (**B**) and (**C**), the solid line shows the decay time under control conditions (no modulators). The dashed line shows the decay time in the presence of 10 nM alfaxalone. The dotted line shows the decay time in the presence of 300 nM alfaxalone. The number of cells was 3–17 for control, drug, or drug combinations. ^#^not significant; *p < 0.05; **p < 0.01; ***p < 0.001.

The concentrations of alfaxalone were selected based on previous data^11^. Exposure to 10 nM alfaxalone produced no effect (τ_D_ = 42 ± 10 ms; n = 9 cells; *p* > 0.9) while the application of 300 nM alfaxalone increased the decay time to 85 ± 26 ms (n = 9; *p* < 0.001). Sample sIPSCs are shown in Fig. 3 and the data are summarized in Fig. 4A.

Bath exposure to micromolar concentrations of propofol increased τ_D_. In the presence of 1 μM or 3 μM propofol, the mean decay times were 82 ± 22 ms (n = 7; *p* < 0.001) or 131 ± 7 ms (n = 4; *p* < 0.001), respectively. At lower concentrations of propofol tested (10–300 nM), the effects were not significantly different from the control value (Fig. 4B).

The propofol-induced increase of decay times was not affected by coapplication of 10 nM alfaxalone with propofol, i.e., at any tested propofol concentration the τ_D_ was not different in the absence and presence of 10 nM alfaxalone (Fig. 4B). In contrast, the combination of 300 nM alfaxalone with 100 nM to 1 μM propofol prolonged the decay time beyond that observed with either drug alone. At 100 and 300 nM propofol the effects of coapplication with 300 nM alfaxalone can be described as supra-additive because exposure to propofol alone did not prolong τ_D_. The results are summarized in Fig. 4B.

Exposure of the cells to 10–1000 nM diazepam increased the decay times of sIPSCs. The τ_D_ was 63 ± 13 (n = 6; *p* < 0.01 vs. control data), 65 ± 16 ms (n = 6; *p* < 0.01) or 87 ± 27 ms (n = 5; *p* < 0.001) with bath-applied 10, 100 or 1000 nM diazepam. The application of 1–3 nM diazepam did not have a significant effect on the decay time course.

Coapplication of 10 nM alfaxalone did not modify diazepam-induced prolongation of τ_D_ at any tested concentration of diazepam (Fig. 4C). In the presence of the combination of 300 nM alfaxalone and 10 nM, 100 nM or 1 μM diazepam, the decay times were greater than in the presence of either drug alone. However, alfaxalone and diazepam alone at these concentrations had significant effects on the decay time. The calculated sums of individual effects were greater than the control-subtracted effects of the drug combinations. For the combination of 10 nM diazepam +300 nM alfaxalone, the control-subtracted τ_D_ was 119 ± 62 ms whereas the sum of control-subtracted effects of individual drugs was 67 ± 31 ms. Similarly, for the combinations of 100 nM or 1 μM diazepam +300 nM alfaxalone, the control-subtracted decay times were 123 ± 37 and 123 ± 35 ms, and the sums of individual drug effects were 69 ± 33 and 91 ± 39 ms, respectively. We used two-way ANOVA^12^ (but see^13^) to determine if the differences between the combined treatments and the sums of individual treatments were significant. Significant interactions were found for alfaxalone and 10 nM diazepam [F(1,34) = 6.91; *p* < 0.013] or 100 nM diazepam [F(1,32) = 13.67; *p* < 0.001]. The interaction failed to reach statistical significance for 1 μM diazepam [F(1,31) = 4.00; *p* = 0.054].

### The effects of alfaxalone on potentiation of recombinant concatemeric α1β2γ2L GABA_A_ receptors by propofol and diazepam

Alfaxalone, propofol and diazepam potentiate the αβγ GABA_A_ receptor activated by low concentrations of the transmitter^14–17^. We examined the effects of the drugs, individually and in combination, on peak responses from concatemeric α1β2γ2L GABA_A_ receptors activated by a low concentration of GABA. Raw current responses were converted to units of open probability, as described in Methods, that were analyzed in the framework of the co-agonist concerted transition model^18,19^.

**Figure 5:**
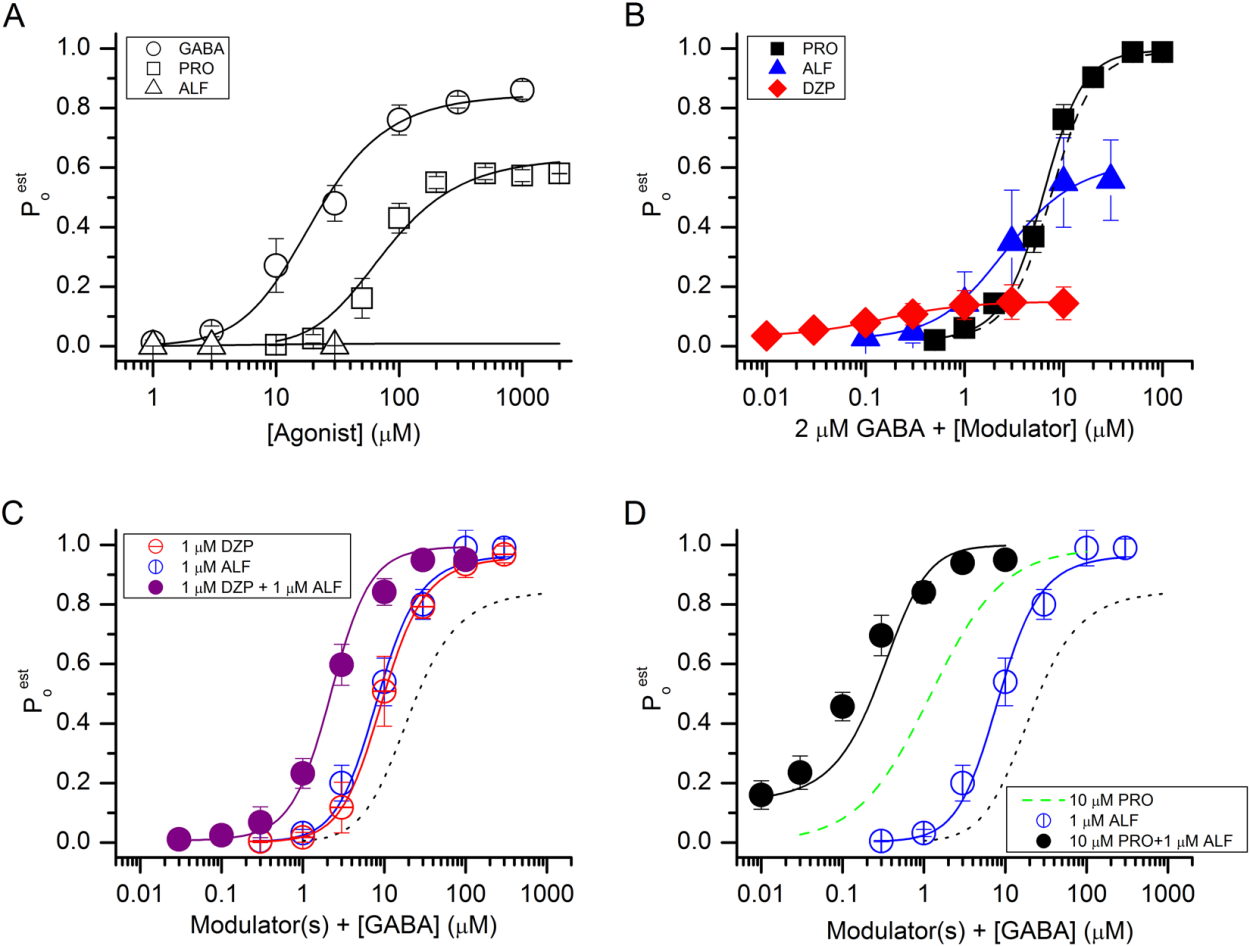
Activation and modulation of concatemeric α1β2γ2L GABA_A_ receptors. (**A**) Direct activation of the receptor by GABA, propofol (PRO), or alfaxalone (ALF). The ordinate shows the estimated open probability (P_o_). The curves for GABA and propofol were generated by fitting Equation (1) to experimental data. Direct activation by alfaxalone produced very small currents (<100 nA at 30 μM). The predicted activation curve for alfaxalone was generated using K_ALF_ and *c*_ALF_ values determined in (**B**). (**B**) Potentiation of GABA-activated receptors by propofol, alfaxalone, or diazepam (DZP). The solid curves were generated by fitting Equation (1) to the data. L was held at (1 − P_o,2 μM GABA_)/P_o,2 μM GABA_. The dashed line shows the predicted propofol-potentiation curve using K_PRO_ and *c*_PRO_ values from (**A**). (**C**) Activation by GABA in the presence of 1 μM diazepam, 1 μM alfaxalone or the combination of diazepam + alfaxalone. The curves for single modulators were generated by fitting Equation (1) to the data. L was held at at (1 − P_o,1 μM ALF_)/P_o,1 μM ALF_ or at (1 − P_o,1 μM DZP_)/P_o,1 μM DZP_ . The open probability for receptors activated by 1 μM alfaxalone or 1 μM diazepam was calculated from the K_ALF_ and *c*_ALF_, or K_DZP_ and *c*_DZP_ values estimated in (**B**). The simulated curve for the combination of diazepam + alfaxalone was calculated using Equation (1) and the K_GABA_ and *c*_GABA_ values from (**A**), and K_ALF_, *c*_ALF_, K_DZP_ and *c*_DZP_ values from (**B**). (**D**) Activation by GABA in the presence of 10 μM propofol, 1 μM alfaxalone or the combination of propofol + alfaxalone. The curve for propofol (green dashed line) is based on data from a previous report^45^. The data for alfaxalone are reproduced from panel C. The simulated curve for the combination of propofol + alfaxalone was calculated using Equation (1) and the K_GABA_, *c*_GABA_, K_PRO_ and *c*_PRO_ values from (**A**), and K_ALF_ and *c*_ALF_ values from (**B**). In (**C**) and (**D**), the black dotted lines show the GABA concentration-response relationship in the absence of modulators (from A). In all panels the data points show mean ± S.D. from at least five cells.

Fitting Equation (1) to pooled estimated open probability (P_o_) data obtained in the presence of 1–1000 μM GABA yielded a K_GABA_ (equilibrium dissociation constant of the closed receptor for GABA) of 35.3 ± 6.6 μM (best-fit parameter ± standard error of the fit) and a *c*_GABA_ (measure of gating efficacy for GABA; see Methods) of 0.0045 ± 0.0004. Analysis of data from receptors activated by 10–2000 μM propofol gave a K_PRO_ (equilibrium dissociation constant of the closed receptor for propofol) of 44.8 ± 8.9 μM and a *c*_PRO_ of 0.04 ± 0.002. The data and the fits are shown in Fig. 5A.

Alfaxalone and diazepam exhibit low efficacy as direct activators of the GABA_A_ receptor. This makes direct determination of the binding and gating parameters in the wild-type receptor technically unfeasible. To determine the binding and gating properties for alfaxalone and diazepam, we measured the potentiation properties of these two drugs coapplied with a low concentration (2 μM) of GABA. In this experiment, GABA acts as a co-agonist that facilitates channel opening and unveils the functional effect of a weak activator.

The data showing potentiation of GABA-activated receptors by alfaxalone, diazepam, or propofol are given in Fig. 5B. Using Equation (1), we estimate that K_ALF_ (equilibrium dissociation constant of the closed receptor for alfaxalone) is 2.9 ± 0.5 μM and *c*_ALF_ is 0.11 ± 0.006. For diazepam, we estimate that K_DZP_ (equilibrium dissociation constant of the closed receptor for diazepam) is 0.17 ± 0.02 μM and *c*_DZP_ is 0.18 ± 0.004. We also recorded potentiation of GABA-activated receptors by propofol, which yielded a K_PRO_ of 52.8 ± 25.7 μM and a *c*_PRO_ of 0.03 ± 0.01. These values are similar to the estimates obtained from direct activation by propofol (44.8 μM and 0.04, respectively), indicating that the actions of GABA and propofol are energetically additive and independent. The data indicate that the potentiating actions of propofol are not due to the drug modifying the affinity of the receptor to the transmitter. Instead, propofol acts as a co-agonist, modifying the open-closed equilibrium. This finding is in agreement with previous reports^20,21^. The activation properties of the receptor in the presence of GABA, propofol, alfaxalone, and diazepam are summarized in Table 1.

**Table 1:** Summary of activation properties.

Agonist	Background agonist	L	K (μM)	*c*
GABA	None	9000	35.3 ± 6.6	0.0045 ± 0.0004
Propofol	None	9000	44.8 ± 8.9	0.04 ± 0.002
Alfaxalone	2 μM GABA	66 ± 36	2.9 ± 0.5	0.11 ± 0.006
Diazepam	2 μM GABA	37 ± 16	0.17 ± 0.02	0.18 ± 0.004

The K (equilibrium dissociation constant of the closed receptor) and *c* (ratio of the equilibrium dissociation constant of the closed receptor to that of the open receptor) values were estimated by fitting Equation (1) to the concentration-response data (Fig. 5). The number of binding sites was held at 2 for GABA, 3 for propofol, 2 for alfaxalone, and 1 for diazepam (see Methods). The value for L was held at 9000 in the absence of a background agonist or at the value calculated from estimated open probability of the background agonist (2 μM GABA) as (1 − P_o,background_)/P_o,background_.

The binding of GABA (to two sites) contributes −6.38 kcal/mol to stabilization of the open state, shifting the open-closed equilibrium by 50,000-fold toward the open state. Propofol contributes −5.70 kcal/mol of stabilization energy. Alfaxalone and diazepam contribute −2.59 kcal/mol and −1.01 kcal/mol, respectively. The relatively weak potentiating effect observed in the presence of diazepam (Fig. 5B) is, in part, due to the receptor containing a single high-affinity binding site for diazepam.

We next determined whether the estimated affinity and efficacy values for alfaxalone and diazepam can be used to accurately predict shifts in GABA-activation curves in the presence of a fixed concentration of a modulator. For that, we measured GABA concentration-response relationships in the presence of 1 μM alfaxalone or 1 μM diazepam (Fig. 5C). The data were analyzed using Equation (1) where the value of L was modified to reflect background activity due to direct gating by the modulator. Since direct gating by 1 μM alfaxalone or diazepam could not be reliably measured (P_o_ < 0.001), the level of activity (P_o,background_) in the presence of alfaxalone or diazepam was calculated using Equation (1) and the K and *c* values for alfaxalone and diazepam determined in the presence of GABA (Fig. 5B).

The estimates for K_GABA_ and *c*_GABA_ obtained in the presence of 1 μM alfaxalone were 39.5 ± 17.5 μM and 0.0046 ± 0.0016, and in the presence of 1 μM diazepam 40.8 ± 5.8 μM and 0.0047 ± 0.0005. These values are remarkably similar to the K_GABA_ and *c*_GABA_ estimates obtained in experiments conducted in the absence of modulators (35.3 μM for K_GABA_ and 0.0045 for *c*_GABA_) thereby indicating that the effects of alfaxalone and diazepam on GABA-activated receptors can be accounted for by independent and additive energetic contributions to the open-closed equilibrium. We attempted fitting the curves to a full co-agonist model^18^ with no constraints on K and *c* for either agonist. However, the fits did not converge, possibly due to poorly defined responses at low GABA concentrations.

Finally, we examined the effect of the combinations of 1 μM diazepam +1 μM alfaxalone and 10 μM propofol +1 μM alfaxalone on the GABA concentration-response relationship. These drug combinations tested the energetic independence and additivity of the agonists in the three-drug mixtures of GABA + diazepam + alfaxalone and GABA + propofol + alfaxalone. The predicted P_o_ curves (Fig. 5C,D), generated assuming that each agonist independently and energetically additively contributed to channel opening, are in good agreement with the experimental data points.

Overall, the data indicate that in the agonist combinations of GABA ± diazepam ± alfaxalone and GABA ± propofol ± alfaxalone, each drug acts independently and energetically additively to stabilize the open state of the concatemeric α1β2γ2L GABA_A_ receptor. In the framework of the state function of the co-agonist model (Equation 1), each drug in these agonist combinations alters the value of L with no effect on the affinity (K) or efficacy (*c*) of the others. We note that energetic additivity manifests as apparent synergy because, at least at low drug concentrations, the response to the drug combination is greater than the sum of individual responses^22^.

## Discussion

Propofol, diazepam and alfaxalone potentiate transmitter-elicited current responses from the GABA_A_ receptor^14–17^. Additionally, propofol is an efficacious direct activator of the receptor^20,21,23^. The GABAergic activity of these compounds underlies their sedative and anesthetic effects^24–26^. The goal of this study was to establish whether coapplication of the neuroactive steroid alfaxalone modifies the actions of propofol and diazepam in different assays aimed at measuring GABAergic inhibitory activity. Specifically, we determined the effects of combining alfaxalone with propofol or diazepam on sedation-like behavior in mice by analyzing time spent at rest and loss-of-righting during an activity test conducted over the 30-min post-injection period. The doses of the drugs were selected to elicit minimal effect when administered alone. The behavioral endpoints were correlated with the effects of combining alfaxalone with propofol or diazepam on the decay time properties of sIPSCs from hippocampal neurons, and on peak current responses from recombinant concatemeric synaptic-type α1β2γ2L GABA_A_ receptors.

We report that co-administration of alfaxalone with either propofol or diazepam in mice greatly amplifies effects on sedation-like behavior. Intraperitoneal injections of the combinations of propofol + alfaxalone and diazepam + alfaxalone resulted in significantly enhanced levels of time spent at rest, compared to mice exposed to a single drug or saline-control treatments. Analysis of behavioral performance over the post-injection test session showed that both combinatorial drug treatments involving alfaxalone produced fast-acting changes in sedation-like behavior such that they each increased time at rest by the first 5-min time block compared to the single drug treatment or saline control groups. Analyzing behavioral responses over the post-injection time also revealed that the diazepam + alfaxalone treatment produced robust increases in rest times throughout the entire 30-min session relative to the single drug/saline groups, while the propofol + alfaxalone administration produced a shorter period of discernible effects, which were limited to the first 20 min post-injection.

Diazepam is often used as an anxiolytic whereas sedation is an undesired side effect. The data, as evaluated by the time at rest, show that co-administration of diazepam and alfaxalone increases the sedative effect of diazepam alone. Our study was not designed to test the anxiolytic effects of the drugs and drug combinations, and the potential clinical usefulness of diazepam + alfaxalone remains to be determined in future studies.

Co-administration of propofol and alfaxalone resulted in loss-of-righting in 10/12 animals whereas none of the animals administered either drug alone or saline experienced loss-of-righting. At the doses selected the combination of diazepam + alfaxalone resulted in loss-of-righting in only 2/10 mice. The difference in the effects of propofol vs. diazepam when administered in combination with alfaxalone in producing loss-of-righting may be attributable to the differences in GABA_A_ receptor subunit selectivity of the drugs, and the mediation of certain sedation-related behaviors by particular subtypes of the receptor. Benzodiazepines, such as diazepam, are selective for GABA_A_ receptors containing a γ subunit^27^ which are predominantly localized to synapses^28^. Propofol and neuroactive steroids modulate all heteromeric GABA_A_ receptor subtypes, including δ subunit-containing extrasynaptic receptors, and are effective anesthetics. A low dose of alfaxalone can only enhance the actions of diazepam at receptor subtypes that are modulated by benzodiazepines and can thus enhance diazepam’s anxiolytic/sedative effects. In contrast, alfaxalone can enhance the actions of propofol at all GABA_A_ receptor subtypes and thus enhances both its sedative and anesthetic effects.

The effects of combinations of alfaxalone with propofol on sIPSCs were supra-additive at several concentration combinations where propofol applied alone had no effect on τ_D_ but significantly enhanced the ability of alfaxalone to modulate the decay time course. For diazepam + alfaxalone, the effects on τ_D_ were greater for the combination than for either drug applied alone, however, each drug applied alone produced a significant effect.

In two-electrode voltage clamp recordings, we employed quantitative analysis based on the co-agonist concerted transition model^18^ to determine the binding and gating properties for GABA, propofol, alfaxalone, and diazepam. The major finding is that the effects of the drugs can be accounted for by independent and additive changes in free energy. The functional effects of the combinations of GABA ± propofol ± alfaxalone and GABA ± diazepam ± alfaxalone could be accurately predicted from the individual effects of the drugs on the open-closed equilibrium assuming independent energetic contributions. We note that the observed energetic additivity predicts that the drug combinations exhibit synergy in isobolographic analysis^22^.

The effective concentrations of the GABAergic modulators were similar in neuronal and oocyte preparations. A 2-3-fold prolongation (statistically significant) of the decay times of sIPSCs was observed in the presence of 300 nM alfaxalone, 1 μM propofol or 10–100 nM diazepam. Inhibitory synaptic events in hippocampal cultures are mediated by GABA_A_ receptors comprising α1, α2, β2, β3 and γ2 subunits^29,30^. In two-electrode voltage clamp recordings on concatemeric α1β2γ2L receptors, these concentrations elicited an approximately 2-fold increase in the peak response.

This study adds to the growing body of evidence demonstrating enhanced functional effects in electrophysiological and behavioral assays resulting from exposure to combinations of GABAergic modulators. Anesthetic steroids potentiate the actions of the intravenous anesthetics propofol and etomidate in electrophysiological^8^ and behavioral assays^9,31^. Similarly, coapplication of neuroactive steroids enhances the anxiolytic effect of the benzodiazepine triazolam^32,33^ and the anticonvulsant potency of diazepam against seizures induced by pentylenetetrazol^34^.

In summary, our results show that the GABAergic effects of propofol and diazepam are enhanced in the presence of alfaxalone. Quantitative analysis of electrophysiological responses from heterologously-expressed receptors shows that the effects of drug combinations are accounted for by independent and additive energetic effects of the modulators, which predict curvilinear isoboles of additivity that are typically associated with synergy. Examination of the properties of sIPSCs indicates that combination of alfaxalone with subthreshold concentrations of propofol elicits supra-additive effects on the decay times of sIPSCs. Behavioral tests in mice show that the actions of propofol and diazepam are amplified in the presence of alfaxalone. These are potentially valuable findings. In cases where the two drugs in a pair are linked to different off-target effects, the combinations of drugs enable reduction of the dose of each drug and, while retaining an intended GABAergic output, may lower the off-target side effects.

## Methods

### Behavioral assays

Behavioral assays were conducted on 7-week old male Swiss-Webster mice. The experiments were conducted in accordance with the Guide for the Care and Use of Laboratory Animals of the National Institutes of Health. The protocol for use of mice was approved by the Animal Studies Committee of Washington University in St. Louis (Approval Nos 20150065; 20150076).

A total of 85 mice purchased from Taconic (Hudson, NY) were acclimated to the new housing conditions for at least 1 week prior to behavioral testing. All mice were housed in a 12/12-light/dark schedule with ad libitum access to food and water. Initial experiments were conducted with alfaxalone, propofol, and diazepam to establish doses that appeared to produce sedation based on the results of ambulatory activity (data not shown) and subsequent injections were performed using a presumed subsedative dose of each drug.

For experiments testing the combinatorial effects of alfaxalone and propofol, mice were injected intraperitoneally either with 20 Units of normal saline, 50 mg/kg propofol, 30 mg/kg alfaxalone, or both 50 mg/kg propofol and 30 mg/kg alfaxalone (n = 11–12 mice per group). Immediately following injection mice were placed individually into translucent (47.6 × 25.4 × 20.6 cm) chambers and sedation-related behavior was quantified over a 30-min period using computerized photobeam instrumentation and standard algorithms as previously described^35,36^. The dependent variable for characterizing the sedative effects of drug treatments was time spent at rest, i.e., lack of movement. This was defined by an absence of a change in the status of any floor-level, photocell pairs (either newly cleared or blocked) along the x- and y-axes for at least a 2-s period. No baseline behavioral measures were collected to minimize possible “floor effects” resulting from decreased motor activity accruing from habituation to the testing environment, and thus help increase sensitivity for detecting sedative drug effects. The combinatorial effects of alfaxalone and diazepam were evaluated in the same manner except that mice were injected either with normal saline, 4 mg/kg diazepam, 30 mg/kg alfaxalone, or both 4 mg/kg diazepam and 30 mg/kg alfaxalone (n = 10 per group). The behavioral experiments were conducted within a 3-hr window to minimize changes in baseline activity associated with circadian rhythm.

Animals were also scored for the loss of the righting reflex during activity testing, which was recorded when a mouse spontaneously assumed a supine or prone position for greater than 30 seconds. This criterion avoided spurious beam breaks that would have occurred from an experimenter placing a mouse on its back and recording righting time when a mouse assumed an upright posture on all 4 limbs.

A repeated measures (rm) ANOVA model (SYSTAT 12, Systat Software, Inc., San Jose, CA), containing one between-subjects variable (drug) and one within-subjects variable (time post-injection) was used to analyze the time at rest data. Simple main effects tests were conducted after the rmANOVA to better understand the nature of drug × time interactions. The Huynh-Feldt (H-F) adjustment of alpha levels was utilized for all within-subjects effects containing more than two levels in order to help protect against violations of the sphericity/compound symmetry assumptions underlying this ANOVA model. General normality of the data was assessed by conducting the Shapiro-Wilk test on the times at rest summed across the activity session for the propofol + alfaxalone and diazepam + alfaxalone experiments. Results from this analysis did not reveal any significant departures from normality. Bonferroni adjusted “*p*” values are presented and were computed by multiplying uncorrected “*p”* values by the number of comparisons that were conducted for a given analysis. Probability values of *p* = 0.0000 are listed as *p* < 0.00005.

### Hippocampal neurons, recordings and analysis of sIPSCs

Cultures of hippocampal neurons were prepared from rat brains as described previously^11,37^. The experiments were conducted in accordance with the Guide for the Care and Use of Laboratory Animals of the National Institutes of Health. The protocol for use of rats was approved by the Animal Studies Committee of Washington University in St. Louis (Approval No. 2015019).

Synaptic currents were recorded from neurons cultured for 9–14 days. Coverslips with cells were transferred to a 60 mm dish containing 5 ml of bath solution. The bath solution contained (in mM): 140 NaCl, 5 KCl, 1 MgCl_2_, 2 CaCl_2_, 10 D-glucose, 10 HEPES (pH adjusted to 7.4 with NaOH). In some cases, 5 µM CNQX and 25 µM DL-APV were added to bath to block glutamate receptor activity. In other cases, sIPSCs were distinguished from sEPSCs by difference in decay properties^38^.

Drugs (propofol, diazepam, and/or alfaxalone) were added to the bath at the indicated concentrations at least 10 min before recording^39^. Each coverslip with neurons was exposed to a single drug or drug combination, to avoid technical artifacts due to incomplete washout of these lipophilic compounds from the cells. Control recordings (not shown) indicate that the sIPSCs are abolished by bath exposure to bicuculline and picrotoxin.

The pipette solution contained (in mM): 140 CsCl, 4 NaCl, 4 MgCl_2_, 0.5 CaCl_2_, 5 EGTA, 10 HEPES (pH adjusted to 7.4 with CsOH). Neurons were clamped at −70 mV. Pipette resistance was 4–5 MΩ. Series resistance was compensated to 75–85% in most recordings. All experiments were done at room temperature.

Currents were amplified with an Axopatch 200B amplifier (Molecular Devices, Sunnyvale, CA), low-pass filtered at 1 kHz and digitized with a Digidata 1322 A interface (Molecular Devices) at 5 kHz. The detection and analysis of synaptic currents were conducted using pClamp 10 software (Molecular Devices) as described previously^11^.

The decay time courses of sIPSCs were characterized by determining the 100-to-25% decay time. We used this parameter rather than fitting the decay time course to sums of exponentials because of variability in the numbers of exponentials required for adequate fit in the presence of high concentrations of steroid and propofol. The data are expressed as mean ± S.D. (number of cells). The statistical analysis on decay times of sIPSCs was conducted using ANOVA with Dunnett’s correction (Stata/IC 12.1, StataCorp, College Station, TX).

### Expression of recombinant GABA_A_ receptors and two-electrode voltage-clamp recordings from *Xenopus* oocytes

Oocytes from *Xenopus laevis* (purchased from Xenopus 1, Dexter, MI) were used as the expression system for recombinant GABA_A_ receptors. Oocyte harvest and animal handling were carried out in accordance with the Guide for the Care and Use of Laboratory Animals of the National Institutes of Health. The protocol for use of frogs was approved by the Animal Studies Committee of Washington University in St. Louis (Approval No. 20170071).

Electrophysiological recordings were conducted on rat GABA_A_ receptors consisting of β2-α1-γ2L and β2-α1 concatemeric constructs, which have been shown to assemble as βαγβα (counterclockwise, top view)^40^. The generation and functional characterization of the concatemeric receptors have been reported previously^41^. The cDNAs in the pcDNA3 expression vector were linearized by digestion with Xba I (NEB Labs, Ipswich, MA). The cRNAs were produced using mMessage mMachine (Ambion, Austin, TX). Oocytes were injected with a total of 18 ng of cRNA in nuclease-free water (final volume 32 nl) and incubated in ND96 with supplements (96 mM NaCl, 2 mM KCl, 1.8 mM CaCl_2_, 1 mM MgCl_2_, 5 mM HEPES, and the supplements 2.5 mM Na pyruvate, 100 U/ml penicillin, 100 μg/ml streptomycin, 50 μg/ml gentamycin; adjusted to pH 7.4 with NaOH) at 16 °C. Electrophysiological recordings were conducted during the next 2–3 days.

Recordings were conducted using standard two-electrode voltage clamp. The oocytes were clamped at −60 mV. The chamber (RC-1Z, Warner Instruments, Hamden, CT) was perfused with bath (ND96) or bath containing the drugs at approximately 7 ml/min. The current responses were amplified with an Axoclamp 900 A amplifier (Molecular Devices, Sunnyvale, CA), filtered at 10 Hz, and digitized with a Digidata 1320 digitizer (Molecular Devices) at a 50 Hz sampling rate. The traces were subsequently analyzed with Clampfit (Molecular Devices) to determine the peak amplitude of the current response.

Receptor activity was analyzed in the co-agonist concerted transition model framework^18,42^. Raw current amplitudes were converted to units of open probability (P_o_)^43,44^. To that end, the current responses from each cell were compared to the reference response to 3 mM GABA + 50 μM propofol, that was considered to generate a response with a P_o_ indistinguishable from 1^21,45^. For example, exposure to saturating GABA produced a peak response that was 86 ± 8% (n = 9 cells) of the response to 3 mM GABA + 50 μM propofol. Accordingly, the maximal P_o_ for GABA was estimated at 0.86 ± 0.08. This is similar to previous estimates of maximal P_o_ for GABA in the ternary αβγ receptor^21,46,47^. The level of activity in the absence of any GABAergic drugs (P_o_ = 0.00011^22^) was excluded in these calculations.

The binding and gating parameters were determined from fitting the pooled estimated P_o_ data to the following equation^18,48^:

1 $${{\rm{P}}}_{{\rm{o}}}=\frac{1}{1+{\rm{L}}\times {[\frac{1+[{\rm{drug}}]/{\rm{K}}}{1+[{\rm{drug}}]/c{\rm{K}}}]}^{{\rm{N}}}}$$

where K is the closed receptor equilibrium dissociation constant for the drug (GABA, propofol, alfaxalone, or diazepam), *c* is a measure of receptor gating efficacy expressed as the ratio of the equilibrium dissociation constant of the open receptor to that of the closed receptor, and N is the number of binding sites for the agonist (constrained to 2 for GABA^49^, 3 for propofol^21^, 2 for alfaxalone^50^, and 1 for diazepam^51^). Stabilization energy provided by a drug was calculated as NRT × ln(*c*), where RT is the multiplication product of the gas constant and thermodynamic temperature, and other terms are as described above.

The parameter L reflects unliganded or background activity from the receptor. In the absence of any GABAergic agonists, it was constrained to 9000^21,22^. In experiments where a low concentration of one GABAergic drug, i.e., a background drug, was coapplied with a range of concentrations of a second GABAergic agonist, L was calculated as (1 − P_o,background_)/P_o,background_, where P_o,background_ is the open probability of the response to the background drug. In a standard potentiation experiment, where several concentrations of a potentiator (propofol, alfaxalone or diazepam) were coapplied with a fixed low concentration of GABA, the response to GABA alone comprised the background response. Since exposure to 1 μM alfaxalone or 1 μM diazepam did not produce robust current responses, the P_o,background_ for alfaxalone and diazepam were calculated using Equation (1) and the K and *c* values estimated for each drug in the presence of low GABA. Curve-fitting was carried out using Origin v. 7.5 (OriginLab Corp., Northampton, MA).

### Materials

The salts used in buffers, alfaxalone, diazepam, CNQX and DL-APV used in electrophysiological experiments were bought from Sigma-Aldrich (St. Louis, MO). Propofol was purchased from MP Biomedicals (Solon, OH). Stock solution of alfaxalone was made in DMSO at 10 mM and stored at room temperature. Stock solution of propofol was made in DMSO at 200 mM and stored at room temperature. For electrophysiological recordings, stock solution of diazepam was made in ND96 at 30 μM and stored at −20 °C. Dilutions to lower concentrations were made from that solution. The highest final concentration of DMSO was 0.3% (v/v). This concentration of DMSO has been shown to be without effect on currents from recombinant α1β2γ2L GABA_A_ receptors or GABA_A_ receptor-mediated synaptic currents^52,53^.

Pharmaceutical grade reagents were used in all behavioral experiments. Propofol (10 mg/ml, Hospira, Inc., San Jose, CA), alfaxalone (10 mg/ml, Jurox, Inc., Kansas City, MO), diazepam (5 mg/ml, Hospira, Inc.), combinations of either propofol and alfaxalone, or diazepam and alfaxalone, or sterile normal saline (20 Units) were injected intraperitoneally using U-100 insulin syringes. Diazepam was diluted to 0.5 mg/ml with normal saline prior to injection.

### Data availability

The datasets generated and/or analyzed during the current study are available from the corresponding author on reasonable request.
